# Communication and patient participation influencing patient recall of treatment discussions

**DOI:** 10.1111/hex.12515

**Published:** 2016-11-21

**Authors:** Claude Richard, Emma Glaser, Marie‐Thérèse Lussier

**Affiliations:** ^1^ Primary Care Research Team Centre intégré de santé et des services sociaux de Laval Laval QC Canada; ^2^ Faculty of Medicine Université de Montréal Montréal QC Canada; ^3^ Family Medicine and Emergency Medicine Department Faculty of Medicine Université de Montréal Montréal QC Canada

**Keywords:** chronic disease, interpersonal communication, lifestyle discussions, medication discussions, patient participation, primary health care, recall

## Abstract

**Context:**

Patient recall of treatment information is a key variable towards chronic disease (CD) management. It is unclear what communication and patient participation characteristics predict recall.

**Objectives:**

To assess what aspects of doctor‐patient communication predict patient recall of medication information. To describe lifestyle treatment recall, in CD primary care patients.

**Design:**

Observational study within a RCT.

**Setting & participants:**

Community‐based primary care (PC) practices. Family physicians (n=18): practicing >5 years, with a CD patient caseload. Patients (n=159): >40 years old, English speaking, computer literate, off‐target hypertension, type II diabetes and/or dyslipidaemia.

**Main variables:**

Patient characteristics: age, education, number of CDs. Information characteristics: length of encounter, medication status, medication class. Communication variables: socio‐emotional utterances, physician dominance and communication control scores and PACE (ask, check and express) utterances, measured by RIAS. Number of medication themes, dialogue and initiative measured by MEDICODE.

**Main outcome measures:**

Recall of CD, lifestyle treatment and medication information.

**Results:**

Frequency of lifestyle discussions varied by topic. Patients recalled 43% (alcohol), 52% (diet) to 70% (exercise) of discussions. Two and a half of six possible medication themes were broached per medication discussion. Less than one was recalled. Discussing more themes, greater dialogue and patient initiative were significant predictors of improved medication information recall.

**Discussion:**

Critical treatment information is infrequently exchanged. Active patient engagement and explicit conversations about medications are associated with improved treatment information recall in off‐target CD patients followed in PC.

**Conclusion:**

Providers cannot take for granted that long‐term off‐target CD patients recall information. They need to encourage patient participation to improve recall of treatment information.

## 1 Introduction

Chronic diseases (CDs) are increasingly prevalent and contribute significantly to the financial burden of health care.^1^ Patient recall and understanding of pharmacological and lifestyle treatment information received during encounters are key intermediate variables towards better adherence, CD management and improved health outcomes.^2^, ^3^, ^4^, ^5^, ^6^, ^7^, ^8^, ^9^, ^10^

Unfortunately, recall is often faulty. Patients remember as little as a fifth of information discussed and immediately forget 40%‐80% of the content of their medical encounters.^6^, ^11^, ^12^ Factors influencing recall cited in the literature can be classified into three categories: (i) patient, (ii) information and (iii) communication. Patient characteristics include age,^6^, ^12^, ^13^ education, health literacy 6, 12, 14, 15, 16 and anxiety.^6^, ^17^, ^18^ Information characteristics include modality (written vs aural),^6^, ^19^, ^20^, ^21^ structure,^11^, ^22^, ^23^, ^24^, ^25^ number of instructions given^14^, ^26^, ^27^ and encounter length.^13^ Clinicians’ communication skills are also related to recall.^3^, ^6^, ^28^, ^29^ Few studies have examined the effect of patients’ communication skills on their recall of information.

Authors stress the importance of activating patients,^30^, ^31^, ^32^, ^33^, ^34^ especially those suffering from chronic diseases.^35^, ^36^ A few studies aiming to increase patient participation have shown benefits in terms of recall.^37^, ^38^, ^39^, ^40^, ^41^ However, the definition of patient participation is unclear. Many studies equate patient participation with patient question asking.^5^, ^40^ The effect of question asking on recall of information remains equivocal; some studies show no effects,^13^, ^27^, ^39^, ^41^ and others show positive effects^38^ or even negative effects.^13^, ^27^, ^42^

The main objectives of this study were to describe recall of lifestyle and medication treatment information and to assess what aspects of doctor‐patient communication and patient participation predict patient recall of medication information, in off‐target chronic disease primary care (PC) patients. This study is an observational study within a randomized control trial. Results from the randomized control trial concerning communication have been reported elsewhere.^43^

## 2 Methods

### 2.1 Study design

This study is an observational study within a randomized trial. The clinical trial, NCT00879736, was registered with ClinicalTrials.gov, and the protocol received ethics approval from the Institutional Review Board Services (IRB). Informed consent was obtained from physicians and patients. All participants were informed about the confidentiality of their data. There were no transcriptions of audiotapes. All direct identifying information was numerically coded. The clinical trial was a three‐arm parallel design that randomized patients into receiving one of the two communication interventions or usual care. Interventions were delivered either through the web only or combined with a workshop. The main objective was to assess the impact of the educational interventions compared to usual care (no additional material) on doctor‐patient communication. Analysing group effects for medication information recall was not possible because of the quantity of missing data. Thus, this study disregarded original group assignment and reports on patients’ correct recall of information about their chronic disease treatments.

### 2.2 Setting and participants

Patients were recruited from nine urban and suburban community‐based PC practices in Ontario (Canada) between March and December 2009, by the study coordinator. The last outcome data were retrieved from patient charts in September 2010.

Participating family physicians (FPs) were chosen using a convenience sample and were considered eligible if they had been in practice at least 5 years, had a practice orientation towards an adult population, including chronic disease patients, agreed to the audio recording of one visit per participating patient. Once physicians consented, patients were approached by the study coordinator and enrolled if they consented and met the following criteria: (i) 40 years or more of age, (ii) ability to speak English, (iii) comfortable using a computer for routine activities such as regular access to the web and e‐mail, (iv) have a routine follow‐up visit scheduled within 3‐4 months of study enrolment, (v) allow access to their medical records and (vi) have at least one of the following three CDs: hypertension, type II diabetes and/or dyslipidaemia. These CDs were diagnosed by their physicians as not meeting treatment targets set by relevant Canadian guidelines.^44^, ^45^, ^46^ Pregnant patients or those actively treated for cancer were excluded.

### 2.3 Procedures

Patients were recruited and enrolled at an initial visit. Participating FP completed a basic socio‐demographic and practice profile questionnaire at study enrolment. Patients meeting inclusion criteria completed a baseline questionnaire including socio‐demographic and clinical data such as length of FP‐patient relationship.^47^ Three to four months later, they returned for their follow‐up visit with their FP following normal scheduling. This visit was audio‐recorded. Immediately following this visit, patients completed a questionnaire that assessed their recall of treatment information they had just discussed with their FP.

### 2.4 Variables & measures

Predictor and outcome variables that were used in analyses and their sources are described in Table 1.

**Table 1:** Predictor, outcome variables and their source

Variables	Source (Instrument)
Predictor variables	
Patient variables	
Age	Baseline questionnaire
Education	Baseline questionnaire
Number of chronic diseases for which patients were enrolled	Physician questionnaire
Information variables	
Length of encounter	Audiotape (RIAS)
Medication status	Audiotape (MEDICODE)
Medication class	Audiotape (MEDICODE)
Communication variables	
Socio‐emotional utterances	Audiotape (RIAS)
Physician dominance	Audiotape (RIAS)
Communication control	Audiotape (RIAS)
PACE composite score	Audiotape (RIAS)
Dialogue score	Audiotape (MEDICODE)
Initiative score	Audiotape (MEDICODE)
Average number of themes discussed per medication	Audiotape (MEDICODE)
Outcome variable	
Recall of treatment information	Post‐visit questionnaire & Audiotape (MEDICODE)

Audio‐taped encounters were coded with the Roter Interaction Analysis System (RIAS)^48^ and MEDICODE.^49^, ^50^ These two validated coding systems and the following measures are described in detail in Lussier et al.^43^ RIAS ascribes an interaction code to all utterances spoken by the physician and the patient during an entire encounter. Codes fall under two large categories: socio‐emotional and instrumental utterances. Socio‐emotional utterances are types of talk where the participant shows agreement, understanding, empathy, expresses concern, disapproval or reassurance, for example. Instrumental utterances are types of talk where the participant gives information or counsels on medical conditions, lifestyle issues or treatments. RIAS allowed us to estimate encounter length, measure physician dominance and patient communication control scores, the proportion of utterances that were socio‐emotional and a PACE composite score assessing “ask,” “check” and “express” utterances. MEDICODE is a content analysis system that codes communication about medications and medical conditions. MEDICODE codes the presence of discussions related to hypertension, diabetes and dyslipidaemia. Furthermore, discussions of lifestyle treatment, such as diet, exercise, stress management, tobacco and alcohol, were also coded. For medication discussions, six of the ten possible thematic metacategories were judged the most relevant for patients with chronic disease to understand and properly adhere to their medications: medication name, instructions (how to take medication), medication main effect (how it works), adverse effects, adherence and concerns. Themes such as “warnings” and “indications to reconsult” were deemed less pertinent in the context of follow‐up care for long‐term patients, where few prescriptions were new prescriptions. The average number of themes discussed per medication was calculated, giving an indication of the extent of the medication discussion.

Medication status was coded in MEDICODE as (i) active discussed: medications that patients are taking without need for a new script, (ii) renewed prescription: renewing a prescription for an active medication, (iii) new prescription and (iv) other: medications which are discussed but are not currently being taken.^51^ For each theme discussed, MEDICODE also codes for interactions, such as who initiates medication discussions and whether there is a dialogue or a monologue. A dialogue score was calculated using the average level of dialogue on medication themes per medication discussed [0=monologue, 1=dialogue]. An initiative score was calculated in the same way [−1=patient initiative, 0=shared initiative, 1=physician initiative].

Three coders, who were blind to group allocation, received an intensive 1‐month training in both methods and were supervised on a continuous basis during the coding by one of the authors (CR). Inter‐rater reliability was calculated on 10% of encounters. Any discrepancies were resolved through group discussion. Average per cent agreement for RIAS categories was 90%. Mean Kappa value for MEDICODE was .83, showing good agreement between coders.

### 2.5 Recall of treatment information

Immediately following the encounter, patients completed the post‐visit questionnaire. They were asked whether they had discussed hypertension, diabetes or dyslipidaemia, and if so to elicit the name of all the medications they remembered discussing for each CD. Patients were also asked whether they had discussed the five other medication themes described above (yes/no) in relation to the named medication. Patients were asked whether they had discussed changing their diet, doing more exercise, reducing their stress, cutting down or quitting smoking and drinking less alcohol (yes/no). Patients’ answers on the post‐visit questionnaire were matched against the audiotape MEDICODE coding. A recall score was created for medication information, with one point awarded for each correctly recalled medication theme. Patients who did not recall the medication name received a score of zero because the questionnaire was only interpretable if it was clear which medication the patient was referring to. Unintelligible medication names, coded by MEDICODE, were excluded from analysis.

### 2.6 Statistical analyses

Baseline characteristics and descriptive statistics were analysed using chi‐square tests and ANOVAs. A linear mixed model was performed to see what variables predicted recall of medication information. Medication was the unit of analysis clustered within patients. The dependent variable was the combined score of recall of medication information. Variables theoretically related to recall in Table 1 were tested in univariate analyses. Variables significantly related to recall in univariate regressions (*P*<.15) were inserted into the final multivariate regression as fixed effects. All tests used an alpha level of significance of .05.

The sample size used here was based on participants from a randomized control trial (n=221).^43^ Analyses concerning medication information are based on a subset of the sample that had complete medication recall data (n=159). Statistical analyses were performed with IBM SPSS Statistics for Macintosh, Version 20.0. Armonk, NY: IBM Corp.

## 3 Results

### 3.1 Socio‐demographic and clinical characteristics

**Figure 1:**
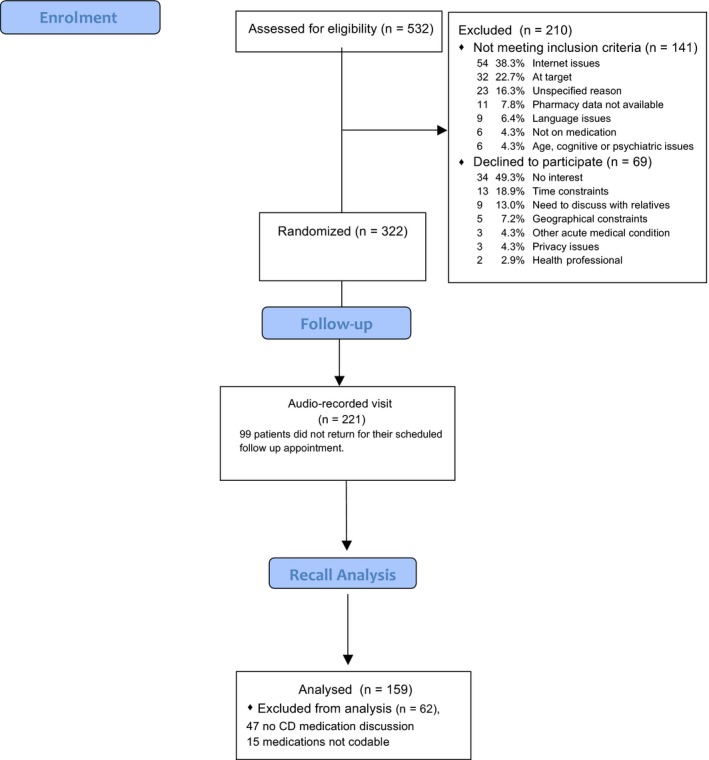
Patient flow diagram. [Colour figure can be viewed at wileyonlinelibrary.com]

A description of the randomization process for the original sample is reported in the primary paper.^43^ Figure 1 describes the consort flow of patients. This study included 221 patients for lifestyle and medical condition descriptions, analysed and described in the original paper, and 159 patients for medication discussions. The 62 excluded patients (47 did not discuss CD medications, and 15 were unintelligible and thus uncodable) did not differ compared to the original 221 on relevant socio‐demographic and clinical characteristics such as education, gender, ethnicity, income, length of relationship with FP, number of visits within the past year and chronic disease profile. However, excluded participants were significantly older than those included here F(1, 219) = 4.49, p = .035, 60.3 (9.1) years vs 57.3 (9.3).

The 18 participating family physicians were 51.2 (6.9) years old, predominantly male (14/18) and in practice for a mean of 25.2 (7.0) years. A majority of these physicians worked in group practices (11 of 18) and saw a mean of 4.5 (1.3) patients per hour (min‐max: 2.5‐8.0). Table 2 shows socio‐demographic and clinical characteristics of patients. Patients were in their late fifties, generally high school educated and white. Most participants knew their physicians for longer than 5 years and had more than four medical visits per year. A third of the sample had more than one active CD. Encounters lasted 10 minutes on average, were physician‐dominated and had a lower proportion of socio‐emotional utterances than information utterances. A small proportion of patients’ utterances were question asking, checking or expressing emotions.

**Table 2:** Socio‐demographic, clinical and encounter characteristics at baseline for 159 patients included in the medication recall analyses

	n=159 patients
Age mean (SD)	57.3 (9.3)
Gender no. (%) male	90 (57)
Education no. (%) ≧high school	116 (73)
Length of relationship with FP no. (%) ≧5 y	118 (74)
Number of visits in past year no. (%) ≧4 visits	105 (66)
Ethnicity no. (%)	
White	118 (75)
Black	9 (6)
Asian	21 (13)
Other	10 (6)
Income $ no. (%)	
<20 000	19 (13)
20 000‐39 000	30 (21)
40 000‐59 999	20 (14)
60 000‐79 999	27 (19)
>80 000	46 (32)
Diagnosis*	
Hypertension no. (%)	95 (61)
Type II Diabetes no. (%)	62 (40)
Dyslipidaemia no. (%)	64 (41)
1 CD diagnosis no. (%)	109 (69)
2 CD diagnoses no. (%)	32 (20)
3 CD diagnoses no. (%)	16 (10)
Encounter characteristics, mean (SD)	
Length, min	10.2 (4.8)
Proportion of socio‐emotional utterances^a^	33.5 (8.7)
Physician dominance score^b^	1.26 (0.44)
Communication control score^c^	0.97 (0.64)
Proportion of PACE‐like utterances^d^	11.0 (5.5)

FP, family physician; CD, chronic disease.

^a^Percentage of total patient‐doctor utterances.

^b^Physician dominance score is a ratio of total physician utterances over total patient utterances. Scores >1 indicate physician dominance.

^c^Communication control score is a ratio of patient control over physician control. Scores >1 indicate patient control.

^d^PACE composite score is a percentage of total patient utterances. It is obtained by summation of patient utterances associated with three communication types of utterances ask, check and express. Further details are given in Lussier et al.^43^

*Some patients were enrolled for more than one diagnosis, so percentages do not equal 100%.

### 3.2 Recall of chronic medical conditions and lifestyle treatment discussions

Recall of whether participants discussed hypertension, diabetes and cholesterol is shown in Table 3. Hypertension, diabetes and cholesterol were discussed in 83.0% (n=183), 60.1% (n=133) and 50.2% (n=111) of the 221 interviews, respectively. Patients recalled approximately 88% of these discussions. Recall of lifestyle treatment is also shown in Table 3. Discussions of stress management, tobacco and alcohol reduction were rare; however, we do not have information allowing us to evaluate the relevance of these discussions (ie known smoking status). Diet and exercise were discussed in about half of the encounters. Recall of these discussions ranged from 42.8% (alcohol) to 70.0% (exercise).

**Table 3:** Recall of chronic disease problems and lifestyle issues discussed, no. (%) (n=221 interviews)*

Recall of problem discussed	
Hypertension	160/183 (87.4)
Diabetes	118/133 (88.7)
Cholesterol	98/111 (88.3)
Recall of lifestyle discussions	
Diet	64/123 (52.0)
Exercise	70/100 (70.0)
Stress	14/24 (58.3)
Tobacco	8/13 (61.5)
Alcohol	3/7 (42.8)

*The denominator indicates the presence of this topic in the interview as coded by MEDICODE. The numerator indicates the patient's response to a yes‐no question of whether they discussed this topic.

### 3.3 Recall of chronic disease medication (CDM) information

The 159 participants analysed for medication discussions discussed a total of 401 chronic disease medications (CDMs). Descriptive statistics regarding medication discussions are shown in Table 4. The majority of medications discussed during the encounter were of two main statuses, either actively being taken or represcribed, consistent with a CD population. Discussions were mostly initiated by physicians and had a low dialogue score. Medication discussions were not extensive in terms of the number of themes discussed. The most often discussed theme besides the name was instructions, mentioned in less than half of the medication discussions.

**Table 4:** Description of medication discussions

	N=401* medication discussions
Medication class no. (%)	
Hypertension	175 (43.6)
Diabetes	122 (30.4)
Cholesterol	104 (25.9)
Medication status no. (%)	
Newly prescribed	17 (4.2)
Active discussed	247 (61.6)
Renewed prescription	65 (16.2)
Other	72 (18.0)
Frequency of theme no. (%)	
Instructions	193 (48.1)
Adverse effects	121 (30.1)
Main effects	139 (34.7)
Adherence	90 (22.4)
Attitudes and emotions towards medications	46 (13.9)
Number of themes discussed, mean (SD)	2.5 (1.2)
Dialogue^a^ mean (SD)	0.28 (0.23)
Initiative^b^ mean (SD)	0.47 (0.63)

*A total of 445 chronic disease medications were discussed. In 15 encounters, 44 of these medications were not identifiable and were excluded from analyses.

^a^Dialogue score ranges from 0 to 1, where 0 is a monologue and 1 is dialogue.

^b^Initiative score ranges from −1 to 1, where −1 is patient initiative and 1 is physician initiative.

**Figure 2:**
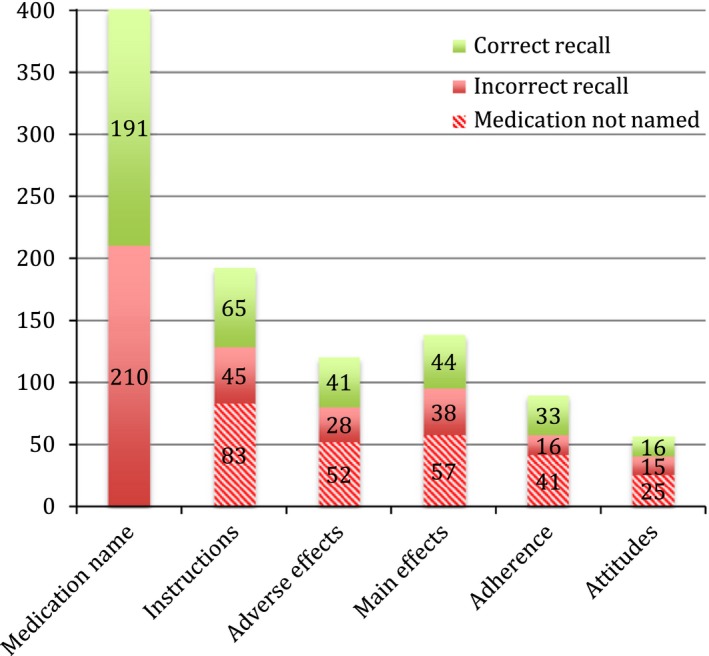
Recall of medication information, no. (%) (n=401 medication discussions, from n=159 interviews)*. *X*‐axis: medication theme. The Y axis denotes the Frequency of theme discussions out of the 401 medication discussions coded with MEDICODE, see Table 4. Green bars are the proportion of patients with Correct recall of the information discussed. Full red bars are patients’ Incorrect recall of information when they were able to elicit the medication name. Patterned red bars correspond to the situation in which patients could not name the Medication discussed in the encounter and thus not indicate discussion of other themes. Patterned red bars are considered incorrect recall.

**Figure 3:**
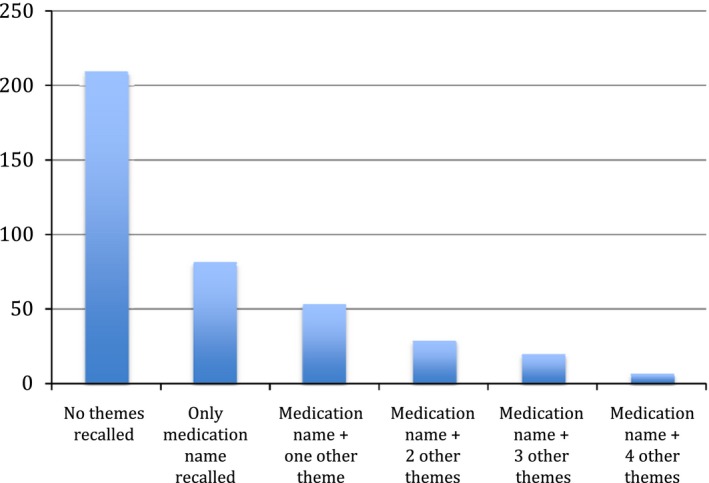
Frequency distribution of medication theme recall. X‐axis: Number of themes correctly recalled. Y‐axis: Instances of theme recall

Figure 2 shows recall of medication themes. Patients were able to correctly elicit the name of the medication for less than half of the 401 medications just discussed. When patients did not correctly identify the medication name, they were coded as incorrectly recalling the five other medication themes (in patterned red). Patients who did not remember discussing a specific medication theme, despite recalling the medication name, are also shown (block red). In green is correct recall. Each theme was approximately correctly recalled a third of the time (eg 65 of 193 for instructions). Figure 3 shows the distribution of the number of themes correctly recalled. Patients recalled less than one theme on average (mean=0.97, SD=1.3, mode=0, median=0).

Table 5 shows the results of the linear mixed model. First, we examined the relationship between the three types of variables (patient, information and communication characteristics) and patient recall in univariate regressions. We found no statistically significant relationship between recall and education or number of CD. For information characteristics, the length of encounter and medication class were not related to recall. Communication characteristics of physician dominance and socio‐emotional utterances were not related to recall.

**Table 5:** Linear Mixed Model for variables influencing correct medication information recall

Variable	Beta estimate (95% CI)	*P* value
Age	−0.014 (−0.029, 0.00)	.057
Medication status discuss/exclude vs active	−0.17 (−0.47, 0.12)	.25
Medication status renew vs active	0.19 (−0.13, 0.51)	.24
Medication status new prescription vs active	0.88 (0.34, 1.42)	.002
PACE	0.013 (−0.011, 0.038)	.29
Communication control	0.085 (−0.12, 0.29)	.41
Presence of Dialogue	0.59 (0.088, 1.08)	.021
Patient Initiative^a^	−0.22 (−0.39, −0.04)	.015
Average number of medication themes discussed	0.48 (0.39, 0.58)	<.001

^a^Initiative scores go from −1 (patient initiative) to 1 (physician initiated).

The multivariate regression showed that while adjusting for relevant patient and information variables, certain aspects of communication were predictors of recall. The extent of discussions (the average number of themes discussed) as well as increased dialogue and patient initiative were all significant predictors of greater medication information recall. The information variables of medication status showed that new prescriptions were better recalled than active prescriptions.

## 4 Discussion

Recall is an important mediating variable for improved treatment adherence and health outcomes.^4^, ^6^, ^11^ This study focused on recall of both lifestyle and pharmacologic treatment information deemed crucial for the optimal management of CD. One of the strengths of this study is its focus on long‐standing CD patients followed in PC and not reaching guideline‐suggested outcomes. Recall was assessed immediately after the medical encounter.

### 4.1 Type of recall measure

The heterogeneity of recall measurements has been recognized and has a major impact on the evaluation of recall.^11^, ^12^, ^52^ The results of our study confirm this. Correct recall of treatment information in this sample ranged from <34% to 88% depending on what was being recalled and how recall was measured. This range is consistent with the bulk of published research.^6^, ^11^, ^12^, ^19^, ^38^, ^52^, ^53^, ^54^, ^55^ Recall was high for a general question such as remembering discussing hypertension. However, recall of specific discussions, such as exercise and diet in relation to the CD, dropped respectively to 70% and only 50% of patients remembering discussing these issues.

Recall scores are higher for yes‐no or multiple‐choice‐type questions, than for open‐ended questions asking patients to elicit information.^56^ This has been replicated in our study, where eliciting the medication name had lower recall scores than yes‐no questions regarding either specific medication themes or lifestyle issues. Considering that yes‐no questions are more easily answered than open‐ended questions, it is of potential concern that only half of patients remember whether or not they discussed diet. This makes one wonder how many would be able to remember specific elements of their diet discussion so important in the management of the three CDs this study focused on.

### 4.2 Recall of medication information

For medication information, the recall score used here was relatively strict, because patients who did not recall medication names were automatically given a score of zero for the other themes. This way of scoring recall may in fact underestimate level of theme medication recall; however, questions were still yes‐no as described above. Patients correctly remembered discussing one medication theme, when 2.5 themes were discussed on average. Patients were able to elicit less than half of the names of medications that they had just discussed with their providers. Medication names are frequently the best recalled items.^22^, ^24^, ^53^, ^57^ A common concept throughout history and across cultures is the idea that knowing the name of something gives one power over it.^58^ Naming, thinking and apprehending reality are intimately related.^59^ Medication names are an important way to establish shared language between patients and physicians and are instrumental in empowering patients to reflect upon, understand and manage their health.^60^, ^61^, ^62^

Discussions of the six themes deemed most important for a patient with CD to properly take and adhere to their medication do not occur frequently. Less than half of medication discussions include instructions about how to take medications, and adherence is mentioned in only about a fifth of discussions. Although it is possible that FPs did not deem necessary to repeat instructions in these actively taken or represcribed medications, this is questionable considering these patients were not at target. Furthermore, patients only remember about a third of these discussions when they actually do take place.

Although concordant with the literature, the low recall scores of medication information are concerning considering the characteristics of our sample. These patients with CD were off target and in long‐term relationships with their FPs. These are precisely the patients who need to be mobilized to properly manage their own care. Patients were generally discussing medications that they were actively taking. Many patients did not even discuss CD medications (47/221) during the audio‐taped follow‐up appointment. These patients were older than the rest of our sample, and it is troubling to see that medication discussions may be eschewed in older patients. Admittedly, one encounter does not capture continuous care. Yet, considering the low rate of recall, discussing relevant medications at each consultation would seem indicated for off‐target CD populations.

### 4.3 Patient participation and medication information recall

Beyond describing recall of treatment information, an aim of this study was to assess which communication and patient participation variables predict medication information recall. There is a need in the literature to be clear about how we define patient participation.^5^, ^63^ In this study, we used two different coding systems with different underlying assumptions about patient participation. RIAS is an interaction‐based coding system. It codes each physician and patient speech act into two metacategories: instrumental and socio‐emotional. Each speech act is then assigned to a *large* content domain such as medical condition, treatment, lifestyle or psychosocial. Participation is classically conceptualized in this system as an aggregate of different types of physician and patient utterances, such as physician dominance and communication control, describing a general pattern unrelated to specific content. Participation can also be defined, as we have done in this study, as PACE‐like interactions reflecting specific information‐seeking and clarification behaviours. MEDICODE, on the other hand, is a content‐based coding system. It first codes for specific content such as medication name, instructions, main effects. Each content element identified is then described in terms of interaction: who, of the two interlocutors, initiated the content, and how much dialogue occurred for that specific theme. Thus, measurement of participation in this second coding system is intimately linked to specific content discussions.

RIAS scores of socio‐emotional utterances, physician dominance, communication control and the PACE score, measuring “ask”, “check” and “express” utterances, were not found to be predictors of patient recall. It is possible that these patient participation measures were not predictors of patient recall because RIAS participation scores reflect a more “global” measure of the *whole* encounter. This does not capture the subtle variations of patient participation in specific discussions of medications.^50^ It is possible to look at RIAS measures in the light of Giddens’ theory of practical and discursive consciousness. RIAS measures, because they reflect interactional styles and processes, can be seen as belonging to the order of practical consciousness. Practical consciousness is knowledge that is inherent in everyday actions, often unnoticed. Talking in a socio‐emotional way is something that patients and physicians do, without always realizing they are doing it. Discursive consciousness reflects knowledge that is verbalized and is thus often better remembered. Content that has been coded by MEDICODE, for example, can be associated with discursive consciousness and may be more easily remembered.^64^

In this study, greater patient initiative and greater dialogue about medications, measured with MEDICODE, were predictors of patient recall. A known cognitive phenomenon, the “generation effect,” stipulates that active involvement in producing information improves recall compared to passive reception.^65^ Authors suggest that this effect may be due to an egocentric bias and a more accurate monitoring of one's contributions to the production of content.^66^, ^67^, ^68^, ^69^ When there is patient initiative and dialogue, patients produce information and are actively involved in the discussion. This finding lends importance to developing skills in improved information exchange.^70^ Unfortunately, providers infrequently use recall‐promoting techniques that aim to involve the patient, such as “teach‐back”.^10^, ^54^, ^71^

Our sample of patients with CD who were familiar with their FPs benefited from greater extent of information, replicating findings from other studies. The more medication themes were discussed, the better patients recalled medication information. Other studies have found information provision to be an important predictor of recall.^27^, ^42^ However, too much information can overwhelm patients,^23^, ^26^, ^55^ an effect that may be mitigated by an established relationship with FPs. Discussing multiple themes surrounding a treatment deepens and adds density to the conversation. This extensive approach to the discussion can enable more conscious involvement.

Of note, new medication prescriptions were better recalled than active prescriptions, which may explain why Tarn et al.^55^ found recall rates upwards of 80% in their examination of new prescriptions. New prescriptions are accompanied with a greater provision of information than active prescriptions,^51^ which may contribute to their better recall. Furthermore, new prescriptions are distinct compared to active prescriptions for this population, and distinct items are often better remembered (the von Restorff effect).^72^

Despite the above noted importance of information provision, medication discussions were often not extensive. Themes that help patients understand what their medications are and why they should be taking them^51^, ^62^ are seldom discussed. Shared decision making is hailed as the future of medical consultations. However, there cannot be any shared decision making if there is not a sufficient information exchange. Street has described three ways of assessing the quality of information exchanges. One is through message properties, such as the content and form of communication. One is through the process of co‐construction of messages, and the third is through the outcomes of the information exchange.^73^ We have included all three ways of measuring this information exchange, using RIAS and MEDICODE to assess the content and form, using dialogue and initiative scores to assess co‐construction and by assessing recall as the outcome of the information exchange. This study shows the importance of having an information exchange that covers essential information about medications. Furthermore, information cannot simply be passively transmitted, as a physician initiated monologue, but needs to be co‐constructed with the patient.

### 4.4 Generalizability

This study had a heterogeneous multimorbid CD patient sample in long‐term relationships with their FPs. This sample is typical of the adult primary care patient population, compared to most studies, which only focus on single diseases, such as diabetes. We are confident that the observations reported are generalizable to community‐based family physicians. Firstly, physicians were given no instructions beyond the fact that they were participating in a communication study. Their interview schedules were not modified for the study, and the average length of encounters was comparable to Ontario average PC visit lengths. Perhaps less generalizable to this population is the criterion of computer literacy. However, computer literacy is rapidly increasing in age groups of 55‐64, where more than 70% of users access the Internet.^74^

### 4.5 Limitations

This study has several limitations. Firstly, a selection bias cannot be excluded. Participants were chosen according to eligibility criteria for the randomized controlled trial that was being conducted. Furthermore, 62 patients from the original sample (n=221)^43^ were excluded from analyses because they did not discuss CDMs (n=47) or CDMs were not identifiable (n=15). Despite participant loss, most baseline factors remained equivalent. This study is not exempt from a possible Hawthorne effect because consultations were audio‐taped. However, studies have shown that the Hawthorne effect is negligible in doctor‐patient communication research.^75^, ^76^, ^77^, ^78^ In addition, it is possible that this study suffers from misclassification bias. The score used to assess recall was strict and was dependent on a questionnaire that was specifically developed for this study. Questionnaires used to assess recall are often not validated, and further research would benefit from the validation of a measure designed to assess treatment information recall.

A further limitation of this project is that data were collected in 2009‐2010. However, we esteem that there has not been significant changes in the practice of medical interviewing, nor in the practice of website use by patient populations similar to the ones recruited in our sample. Web interfaces similar to what was developed in our project Talking Health Together (THT) do exist. In fact, some of these interfaces are more interactive than what was developed in our project. This suggests that the impact seen in the THT study underestimates the potential impact of similar web tools. We have little reason to believe that the behaviour of patients and physicians currently differs from what was observed during our project. Recent projects using MEDICODE show that doctor‐patient communication is similar to what was described in the original MEDICODE studies and in the THT study.^43^, ^51^, ^79^, ^80^

Patient involvement and engagement in the development of the THT website was indirect. A committee of experts, including clinicians and patient‐clinician communication researchers, was consulted for the development of the intervention content and the trial format. The website interface was iteratively tested with laypersons to adjust the format (font, navigation, etc.) and content of the site. The value of the approach used in THT has been confirmed by results of a validation study of a francophone website « Discutons Santé » (« Lets Discuss Health »). This website was based on THT. Patients were consulted throughout the development of this website, including a pilot test of the penultimate version. The implementation of this website into routine clinical use in primary care is currently taking place, a major practice implication of the THT study. Preliminary results from focus groups show that the website is greatly appreciated by patients.^81^, ^82^

## 5 Conclusion & Practice Implications

The aims of this study were to describe treatment information recall and to assess which communication and patient participation variables are related to medication information recall for off‐target CD patients. Providers cannot take for granted that long‐term off‐target CD patients know and remember treatment information. This is particularly relevant considering the ageing demographics of PC populations. Elderly patients were less likely to discuss their CD medications and had poorer recall when they did. Further research is needed to understand the dynamics between ageing patients, medication discussions and recall. There is a lack of shared language and empowerment in managing one's disease. Encounters with patients with CD need to engage in an explicit conversation about relevant lifestyle modifications and medications.^70^ This conversation should ideally be thorough and engage patients in a true dialogue. Providers and patients need to engage more frequently about treatment information in ways that encourage patients to be active participants in the discussion. Improved discussions may impact treatment information recall. This is an important step towards improved self‐management, adherence and eventually better health outcomes.

## Conflicts of Interest

This project was sponsored by Astra Zeneca Canada. The sponsor had no role in the collection, analysis and interpretation of data; in the writing of the report; and in the decision to submit the manuscript for publication.
